# Plant reproductive strategies and pollinator attributes differ in small-scale habitat heterogeneity

**DOI:** 10.1093/aobpla/plaf052

**Published:** 2025-09-17

**Authors:** Dongzhou Deng, Juanli Chen, Li He, Dawei Li, Dechao Chen, Wuxian Yan, Junpeng Mu

**Affiliations:** Ecological Restoration and Conservation on Forest and Wetland Key Laboratory of Sichuan Province, Sichuan Academy of Forestry Sciences, 18 Xinghui West Road, Chengdu 610081, China; Ecological Security and Protection Key Laboratory of Sichuan Province, Mianyang Normal University, 166 Mianxing West Road, Mianyang 621000, China; Ecological Restoration and Conservation on Forest and Wetland Key Laboratory of Sichuan Province, Sichuan Academy of Forestry Sciences, 18 Xinghui West Road, Chengdu 610081, China; Ecological Security and Protection Key Laboratory of Sichuan Province, Mianyang Normal University, 166 Mianxing West Road, Mianyang 621000, China; Ecological Restoration and Conservation on Forest and Wetland Key Laboratory of Sichuan Province, Sichuan Academy of Forestry Sciences, 18 Xinghui West Road, Chengdu 610081, China; Ecological Restoration and Conservation on Forest and Wetland Key Laboratory of Sichuan Province, Sichuan Academy of Forestry Sciences, 18 Xinghui West Road, Chengdu 610081, China; Ecological Security and Protection Key Laboratory of Sichuan Province, Mianyang Normal University, 166 Mianxing West Road, Mianyang 621000, China

**Keywords:** alpine meadow, bee behavior, reproductive strategies, land restoration, pollination

## Abstract

Habitat variability critically influences plant reproductive strategies and pollinator attributes. However, studies on intraspecific variation in vegetative and floral traits, pollinator attributes, and seed traits remain limited in the context of small-scale habitat heterogeneity, particularly meadows interspersed with sandy patches. On the Tibetan Plateau, discrete sandy patches (some as small as 10 m^2^) occur within alpine meadows. We hypothesized that distinct plant reproductive strategies and pollinator attributes exist between meadows and sandy habitats at a microhabitat scale. To test this hypothesis, we conducted a field experiment to investigate variation in floral traits, pollinator attributes, and seed traits in a Tibetan alpine herb (*Astragalus purpurinus*) across meadow and sandy habitats. Our results show that meadow populations produced fewer nectar-enriched flowers with high sugar concentrations, fewer and larger seeds, and were pollinated primarily by bumble bees. In contrast, sandy-habitat populations produced numerous nectar-poor flowers with low sugar concentrations, more numerous small seeds, and relied on mason bees for pollination. Our results demonstrate that micro-scale habitat heterogeneity drives divergent plant reproductive strategies and pollinator attributes within a single species. These findings reveal novel mechanisms by which small-scale environmental variation shapes reproductive adaptation in alpine ecosystems.

## Teaser

Habitat variability critically influences plant-pollinator interactions. However, we know little about how intraspecific variation in plant reproductive strategies and pollinator attributes responds to small-scale habitat heterogeneity, such as meadows interspersed with sandy patches. To address this, we performed a field experiment in Tibetan meadows and sandy habitats to examine the impact of these differences on floral, pollinator, and seed traits. Our data indicate a notable disparity: meadow populations produced less nectar-abundant flowers and larger seeds, predominantly pollinated by bumble bees. Conversely, populations in sandy habitats produced a higher number of nectar-deficient flowers and smaller seeds, depending on mason bees for pollination.

## Introduction

Climate change and human activity are modifying the habitats of flowering plants. Human-caused habitat heterogeneity can influence various plant reproductive traits, including floral production and display, flower size, the quantity and quality of nectar and pollen, and flowering phenology (Aizen and Vazquez 2006, Jacquemyn *et al*. 2012, Xiao *et al*. 2016, Gargano *et al*. 2017, Librán-Embid *et al.* 2021, Chen *et al.* 2025). Additionally, an increase in habitat heterogeneity leads to changes in pollinator attributes, including total visitation rates, pollinator diversity, and the composition of the pollinator fauna (Aizen and Vazquez 2006, Ekroos *et al.* 2013, Neumüller *et al*. 2020). These disparities in plant traits and pollinator attributes, resulting from habitat heterogeneity, influence plant reproductive strategies (Aguilar *et al*. 2006, Evans *et al*. 2017, Reverté *et al*. 2019). For instance, in resource-abundant habitats, plants produce larger, nectar-rich flowers to attract pollinating insects, thereby increasing seed yield and reproductive success (David *et al*. 2019, Dai *et al*. 2022, Gao *et al*. 2023). In contrast, plants in resource-limited habitats (e.g. low water, poor nutrients) confront significant challenges to survival and reproduction. To maximize fitness and ensure species persistence, they have evolved diverse specialized reproductive strategies. These often involve trade-offs, diverting energy from vegetative growth towards reproductive functions like flower size, pollen production, and seed output (Waser and Price 2016, Mueller *et al*. 2020, Dai *et al*. 2022). The majority of research focuses on the effects of large-scale (regional scale) habitat heterogeneity on plant-pollinator interactions, with only a few studies examining the implications of small-scale habitat heterogeneity (local scale).

At the local-patch scale, small-scale habitat heterogeneity might create distinct micro-niches, potentially influencing plant–pollinator interactions. Recent studies indicate that small-scale habitat heterogeneity can increase field border density (Hass *et al*. 2018). This increased density, in turn, enhances the quality and quantity of floral rewards (e.g. pollen and nectar), increases flower abundance, and promotes pollinator richness and abundance (Hass *et al*. 2018, Battle *et al*. 2021, Berthon *et al*. 2021, Bueno and Iglesias 2021). Consequently, these improvements elevate the diversity of plant–flower visitor interactions and increase plant reproduction. Our previous study shows that small-scale habitat heterogeneity, like changes in soil moisture over short distances, causes plants to evolve specific floral traits (e.g. different flower color and flower size) to entice bees and flies for pollination (Dai *et al*. 2022).

Arid and wet habitats constitute a prominent form of habitat heterogeneity, which in turn affects vegetation traits, floral traits, and seed yields (Tadey *et al*. 2009, Dai *et al*. 2022, Rauschkolb *et al*. 2022). Plants that grow in arid habitats usually evolve traits that help them survive in dry conditions, like having low above-ground biomass and allocating limited energy to nectar and flower production (Li *et al*. 2005, Hassan *et al*. 2017, Kuppler *et al*. 2021). Plants with reduced flower resources are associated with specific insect pollinators, such as arid plants that rely on ants and beetles for pollination (Raine *et al*. 2002, Phillips *et al*. 2018). Furthermore, arid plants often produce a larger number of small seeds that are dispersed great distances by the wind, facilitating the establishment of new habitats (Venable *et al*. 2008). In contrast, plants growing in meadows produce larger, nectar-enriched flowers, attracting more effective pollinators and generating high-quality seeds (Petanidou *et al*. 1999, Gallagher and Campbell 2017, Dai *et al*. 2022). Larger seeds supply seedlings more energy throughout the germination process, which helps them compete with other species and enhance their establishment (Jakobsson and Eriksson 2000). However, most studies on plant reproductive strategies and pollinator attributes in arid and wet habitats focus on large-scale effects, with limited attention to small-scale effects. There is a scarcity of studies investigating how alterations in the reproductive strategy of the same species respond to small-scale changes in their habitats.

The Tibetan Plateau is characterized by habitat heterogeneity, which includes sandy habitats due to climate change and human activities (Xue *et al*. 2009, Li *et al*. 2021). The Tibetan alpine meadow contains interspersed sandy habitats, with individual sandy patches measuring under 10 m². Habitat conditions alter significantly over short distances (Gao *et al*. 2016, Wang *et al*. 2023), resulting in variations in plant traits and pollinator attributes. In addition, the low temperature and high precipitation contribute to significant species diversity in the meadows, comprising sedges, grasses, and forbs, including Asteraceae, Gentianaceae, and Fabaceae (Mu *et al*. 2014, Hu *et al*. 2024). However, species diversity in sandy habitats is low (Wang *et al*. 2023, 2025). Most research in this region focuses on soil physical and chemical properties and community diversity (Wang *et al*. 2023, 2025). There is limited understanding of the mechanics of species adaptation, especially how the same species acclimatizes to different habitats over short distances. To our knowledge, limited data have examined plant reproductive strategies within the same species across alpine meadows and alpine sandy habitats at a small scale. This study addresses three questions: (i) Do the flower traits of the studied plant populations differ in response to small-scale habitat heterogeneity? We predicted that plants from the alpine meadow would produce nectar-rich, high-quality flowers, whereas plants from sandy habitats would produce nectar-poor, low-quality flowers. (ii) Do pollinator richness and visitation rates differ under small-scale habitat heterogeneity? Our prediction was that bumble bees would be attracted to flowers in alpine meadows, while mason bees would be attracted to those in sandy habitats, reflecting their preferences for distinct flower traits. (iii) Do the seed traits of the studied populations differ between Tibetan alpine meadows and sandy habitats on a small scale? We predicted that populations from the alpine meadows would produce few, large seeds, whereas populations from the alpine sandy habitats would produce numerous small seeds.

This study was conducted on the alpine meadows on the Zoige Plateau in China, where sandy habitats are interspersed within the meadow. Previous studies indicate that the levels of soil moisture and soil nutrient levels are higher in alpine meadows compared to the sandy habitats (Mu *et al*. 2014, Dai *et al*. 2022, Wang *et al*. 2025). We conducted field experiments using a Tibetan alpine herb (*Astragalus purpurinus*) to examine the effects of small-scale habitat heterogeneity on plant reproductive strategies and pollinator attributes. We measured the quantity and quality of nectar, the number of flowers per plant, the number of inflorescences per plant, plant height and above-ground biomass, seed mass and the number of seeds per plant, reproductive allocation, and pollinator richness and visitation rates between alpine meadows and alpine sandy habitats. These findings will elucidate the mechanisms through which small-scale habitat heterogeneity influences plant reproductive strategies and pollinator attributes.

## Materials and methods

### Study site and species

The study sites were located in the Zoige Plateau, within the eastern Tibetan Plateau (32°20′–34°05′N and 101°36′–103°55′E). The elevation is between 3400 and 3900 m. This climate is characterized by prolonged winters and short summers. The mean annual temperature is recorded at 1.73°C, with total annual precipitation of 756 mm. The yearly average relative humidity is ∼69%, and there are no definitive frost-free periods. The primary soil types in the region consist of subalpine meadow soil and mountain meadow soil (Mu *et al*. 2014, Wang *et al*. 2023).

The species richness differs between meadows and sandy habitats. Meadows encompass over 30 species per m^2^, including *Saussurea nigrescens*, *Anemone trullifolia*, *Deschampsia caespitosa*, *Festuca ovina*, *Blysmus sinocompressus*, *A. purpurinus*, and *Kobresia setchwanensis* (Dai *et al*. 2022, Hu *et al*. 2024). Vegetation coverage surpasses 95%, with an average plant height of around 30 cm (Dai *et al*. 2022). However, sandy habitats have 10 species per m², including *Kobresia myosuroides, Elymus dahuricus Turcz*, *Elymus sibiricus*, *Heracleum hemsleyanum, Acanthocalyx alba, Aconitum gymnandrum,* and *A. purpurinus* (Huang *et al*. 2023, Wang *et al*. 2023).

**Figure 1.**
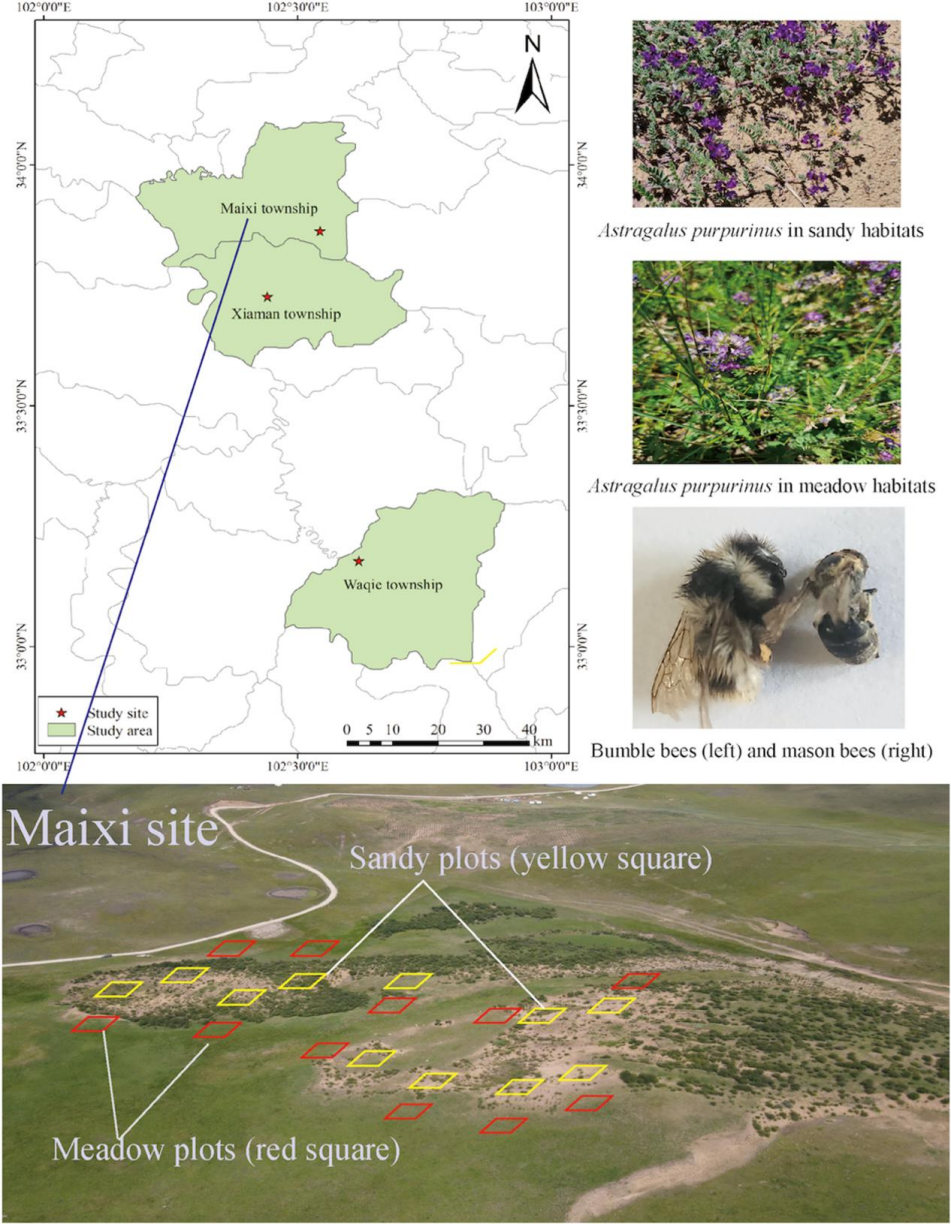
The figure shows the study sites and plant population and bee species of alpine meadows and sandy habitats. There were three sites, and each site included 10 meadow (red square) and 10 sandy plots (yellow square). Each plot was 2 m × 2 m. Totally, there were 3 sites, 60 plots, and 180 plants. Photo Credits: J. Mu

We selected three study sites in the Zoige Plateau (Fig. 1). Site 1 was located in Waqie (33°10′40.8″N 102°37′11.0″E), Site 2 was located in Maixi (33°51′46.8″N 102°32′37.3″E), and Site 3 was located in Xiaman (33°43′36.3″N 102°26′23.8″E). Each site comprised two subsites: meadows and sandy habitats. Meadows are solely grazed by cattle throughout winter, while the sandy regions remain ungrazed (Wang *et al*. 2023, Mu *et al*. 2014). Each subsite comprised 10 plots. The plots were 2 m × 2 m. In total, there were 3 sites, 6 subsites, and 60 plots.

*Astragalus purpurinus*, a perennial herbaceous species within the Fabaceae family, was used as the study species. It typically thrives at elevations between 2500 and 4000 m. Plants reach a height of 30–50 cm, producing 10–20 flowers per inflorescence. This species becomes green in mid-May each year and undergoes senescence in mid-September. Plants typically bloom from late-June to August and bear fruit from July to September.

### Measurement of vegetation and flower traits

During the peak of the flowering period in 2022, the vegetative and floral traits of each plant were assessed, including plant height, nectar volume per flower, nectar concentration, number of flowers per inflorescence, number of inflorescences per plant, pollinator richness, and visitation rates. Upon fruit maturation, we collected fruits, stems, and leaves. The stems and leaves were dried at 65°C for 48 hours. We determined the reproductive allocation by dividing the seed mass by the total dry mass of stems and leaves for each plant. The above-ground biomass was the sum of leaf mass plus stem mass. Each plot contained 3 plants, and each subsite had 30 plants, totaling 180 plants of *A. purpurinus*.

We randomly selected three flowers from each plant. Subsequently, we encased each flower in cylindrical metal netting for 24 hours to exclude pollinators before quantifying the nectar volume and concentration per flower (Real and Rathcke 1991). We quantified the nectar volume per flower using 1- or 2-µl micropipettes. A hand refractometer (Eclipse, Stanley and Bellingham, Basingstoke, UK) with a precision of 0.5% was used to measure nectar concentration (Brix scale). The measurement was conducted on a total of 540 flowers.

We assessed the number of flowers per inflorescence and the number of inflorescences per plant during the flowering period. We quantified the total flower number per plant by multiplying the number of flowers per inflorescence by the number of inflorescences per plant daily at noon. Flowers that were counted were marked with strings to ensure that only new flowers were counted each day. We used a rule to measure plant height, defined as the height of the photosynthetic tissue from the ground surface (Heady 1957). Because the calyx tube length is associated with pollinator diversity (Dohzono *et al*. 2004), we determined the average flower size per plant by measuring the calyx tube length (cm) of three flowers.

### Measurement of richness and visitation rates of pollinators

We netted bumble bees and mason bees, which we could not identify in the field (McFrederick and LeBuhn 2006). The specimens were identified in the laboratory. All samples were collected during the peak of the flowering period.

We measured the pollinator visitation rate as the number of pollinator visits per flower each hour. We initially recorded the number of flowers for each sample plant. Observers documented pollinator visitation to the flowers from a distance of three meters. We evenly distributed the observation intervals throughout the day, from 09:00 to 17:00. During each hourly interval, a single observer scrutinized each distinct plant for 2 minutes from a stationary position. With six observers, each plant was observed twice per hour. Observations recorded the number of flowers per plant that received bee visits during each observation period (Vaudo *et al*. 2014). We conducted the observations exclusively on sunny days, repeating them five times for each plant in 2022. We monitored each individual plant for a total of 90 minutes per year. We calculated the visitation rates (R) per flower per hour by dividing the total number of visits (Nv) per hour by the number of flowers (Nc), which we represented as R = Nv/Nc (Arroyo *et al*. 1985).

### Measurement of seed trait with pollinators and hand-supplemented pollen

We meticulously picked, counted, and weighed each fruit (from August to September) to the nearest 0.001 g upon maturation. We regarded empty seeds as an indication of failure. We defined ‘seed yield’ as the mass of seeds produced per flower. The average seed mass (mg) was calculated by dividing the total seed mass per flower by the number of seeds per flower.

In 2022, we conducted hand-supplemental pollen experiments because pollinator density and foraging rates influenced seed yield during the peak of the flowering season. For hand-supplemented pollen experiments, one flower was selected from each sampled plant. This included 30 plants in alpine meadows and 30 plants in alpine sandy habitats within each subsite. We collected fresh pollen from the anthers of 30 distinct plants for manual supplementation once stigmas matured. Using a fine paintbrush, we carefully transferred the pollen grains to the stigmas of flowers. Pollen transfer occurred exclusively among plants within the same populations. After the fruits ripened, we collected the pods, measured the seed number and seed mass, and calculated seed set as previously described.

### Measurement of soil temperature, moisture, and nutrition

To test the soil moisture and temperature between two habitats, we used Hobo Data Loggers (Pro V2) to monitor the daily soil temperature and moisture levels of the soil throughout the experiment. After collecting all the plant samples, soil samples were collected from 0 to 20 cm depth using a 20 cm diameter soil auger. We analyzed total soil nitrogen, phosphorus, and carbon in the laboratory (Wang *et al*. 2023).

### The effects of nectar traits or community composition on pollinator richness and visitation rates

We conducted a pot experiment to evaluate the impact of community composition on pollinator richness and visitation rates in 2023. During the peak of the flowering period, 30 individuals of *A. purpurinus* were randomly selected from meadows and sandy habitats, respectively. All plants were transferred into pre-prepared containers containing indigenous soil. The pots (each 27.5 cm in diameter and 30.0 cm in height) were sterilized with 70% ethanol (Gao *et al*. 2023). Each pot had a single plant. We categorized the pots into two groups, including 15 pots from meadows and 15 pots from sandy habitats. One group was placed in alpine meadows, and the other group was placed in sandy habitats. We assessed pollinator diversity and visitation rates using the aforementioned approaches. The observations were performed solely on sunny days. In each hourly interval, a single observer examined each plant for 4 minutes from a fixed location. Each individual plant received a total of 96 minutes of field observations annually.

We performed an experiment to assess the impact of the quantity of nectar supply on pollinator richness and visitation rates in alpine meadows versus sandy habitats. During the peak of the flowering season in 2023, we randomly selected 10 individuals of *A. purpurinus* from both meadows and sandy habitats. We randomly selected ten flowers from each plant. Prior to the nectar addition experiment, we used capillaries to collect all nectar previously secreted from the flowers. We added 0 (purified water), 0.1, 0.2, 0.3, and 0.4 µl of a 40% sucrose solution to flowers 1 through 5, while providing equivalent volumes of a 20% sucrose solution to flowers 6 through 10. We delivered the sucrose solution into the spur's tip using a 10 µl microsyringe (Johnson *et al*. 2004). We evaluated the pollinator visitation rate using the previously described methods. Recognizing that bumble bees and mason bees prefer different nectar concentrations (Cnaani *et al*. 2006, Ahrenfeldt *et al.* 2019), two separate nectar concentrations (40% and 20%) were used throughout the experiment.

To assess whether nectar quality affected pollinator attributes, we conducted a nectar addition experiment using different sucrose solution concentrations at Site 1 in 2023. We randomly selected 10 individuals of *A. purpurinus* from both meadows and sandy habitats during the peak of the flowering season. We randomly selected 6 flowers from each plant. Before the nectar addition experiment, we used a 10 µl microsyringe to collect all previously secreted nectar from the flowers. Flowers labeled 1 to 6 received 0.2 µl of sucrose solutions at concentrations of 0%, 10%, 20%, 30%, 40%, and 50%, respectively. We observed that the base of the calyx tube is where plants store their nectar. Hence, we added the nectar to the base of the calyx tube. The sucrose solution was delivered into the spur's tip using a 10 µl microsyringe (Johnson *et al*. 2004). We evaluated the pollinator visitation rate using the previously described methods.

### Statistical analysis

In this study, the data for each trait were averaged for each plant prior to analysis. All statistical analyses were conducted using R (R version 4.4.1; R Core Team 2024).

We used Wilcoxon rank-sum tests to assess the effects of small-scale habitat heterogeneity on vegetation and floral traits, pollinator visitation rates, and seed traits. This non-parametric approach, suitable for comparing differences between two independent groups, was implemented using the Wilcox test function from an R package. Specifically, vegetation and floral traits (e.g. plant height, nectar volume, and number of inflorescences per plant) and reproductive success metrics (e.g. seed set and fruit-to-flower ratio), pollinator visitation rates, and species richness were all compared across habitat types using this test.

Principal coordinate analysis (PCoA) based on Bray–Curtis distance (functions cmdscale, ordiplot, and ordiellipse from the Vegan package) was used to examine the patterns in soil properties, vegetation and flower traits, and pollinator visitation rate across alpine meadows and alpine sandy habitats (Oksanen *et al*. 2025). The two primary components that most effectively explained the variation in community structures were used to construct the PCoA plots. Z-score standardization was performed prior to PCoA.

We analyzed the effects of soil temperature and soil properties on plant traits using generalized linear mixed models (GLMMs). The fixed effects encompassed soil temperature and soil properties (soil moisture, TN, TP, and TC), while the plots and the plants were considered random effects. First, we modeled the flower number, inflorescence number, and seed number with a Poisson model (log-link) using Laplace approximation to estimate parameters. Second, we modeled the seed set with a negative binomial model (logit-link) using Laplace approximation to estimate parameters. Finally, we modeled the nectar volume and concentration and flower size with a Gaussian model (logit-link) using Laplace approximation to estimate parameters.

We used random forest models analysis using the ‘randomForest’ R package (Rigatti 2017) to ascertain the principal parameters affecting pollinator visitation rates. The principal parameters considered included nectar volume and concentration, flower size, plant height, and total flower number per plant.

We analyzed correlations among all variables using the R package ‘linkET’. Relationships between pollinator visitation rates and nectar rewards (such as nectar volume per flower and nectar concentration), between seed set and pollinator visitation rates, as well as between seed mass and seed number, were analyzed using the method of least squares.

## Results

### Do the soil properties vary across the habitats?

Soil properties differed between alpine meadows and sandy habitats. Alpine meadows had higher total nitrogen (*W* = 8100, *P* < 0.001, *n* = 90), phosphorus (*W* = 8100, *P* < 0.001, *n* = 90), and carbon (*W* = 8100, *P* < 0.001, *n* = 90) compared to alpine sandy habitats (see Supplementary Figure S1). Compared to alpine sandy habitats, meadow habitats had higher soil moisture (*W* = 8100, *P* < 0.001, *n* = 90) but lower soil temperature (*W* = 0, *P* < 0.001, *n* = 90; see Supplementary Figure S1).

### Do the vegetative and flower traits and the relative coverage of *A. purpurinus* vary across the habitats?

**Figure 2.**
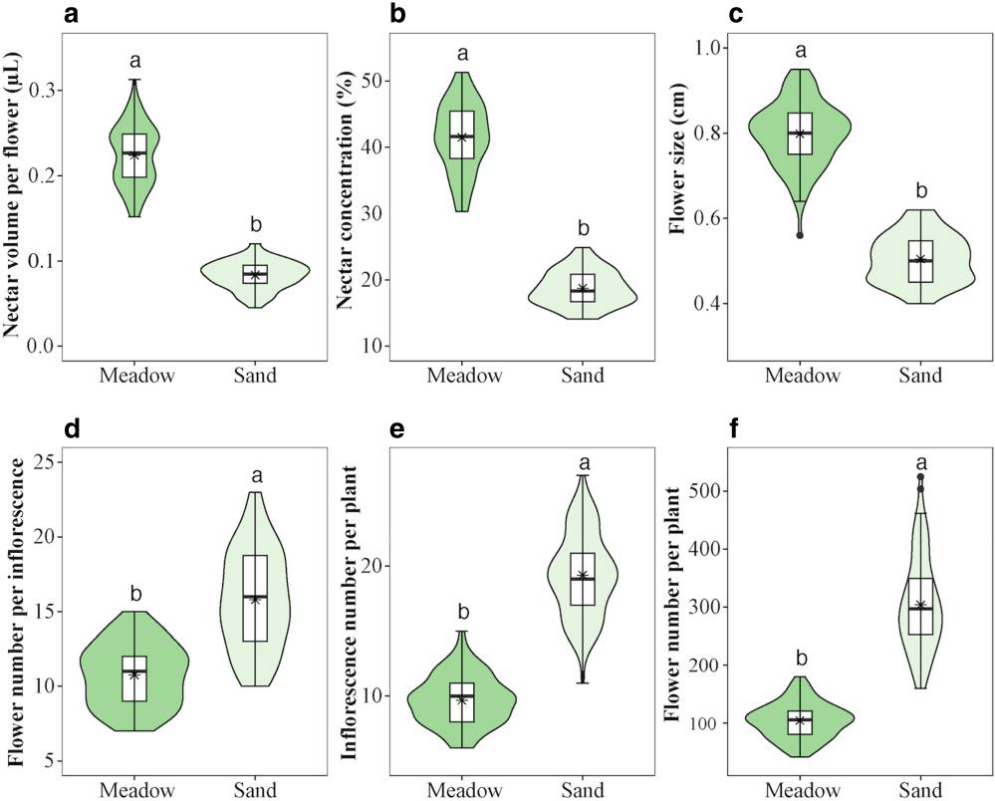
The violin plots show nectar volume per flower (a), nectar concentration (b), flower size (c), number of flowers per inflorescence (d), number of inflorescences per plant (e), and number of flowers per plant (f) of *Astragalus purpurinus* between alpine meadows and alpine sandy habitats. Violin plots show the density (width), interquartile range (hinges), and 1.5 times the interquartile range (adjacent lines). The line within the box represents the median of the responses, and the black star within the box represents the mean of the responses. Diﬀerent letters above the boxes denote signiﬁcant diﬀerences among treatments (*P* < 0.05).

Small-scale habitat heterogeneity significantly influenced vegetative and floral traits (Supplementary Figure S2). Plants in alpine meadows exhibited greater plant height (*W* = 8100, *P* < 0.001, *n* = 90), higher nectar volume per flower (*W* = 8100, *P* < 0.001, *n* = 90), higher nectar concentration (*W* = 8100, *P* < 0.001, *n* = 90), and larger flower size (*W* = 8083, *P* < 0.001, *n* = 90) compared to those in alpine sandy habitats (*W* = 8100, *P* < 0.001, *n* = 90; Fig. 2). However, plants in sandy habitats exhibited a greater number of flowers per inflorescence (*W* = 859, *P* < 0.001, *n* = 90) and a greater number of inflorescences per plant (*W* = 31.5, *P* < 0.001, *n* = 90; Fig. 2).

Small-scale habitat heterogeneity significantly influenced the relative coverage of *A. purpurinus* and the average community height. Plants in alpine meadows had lower relative coverage (about 5%) in contrast to alpine sandy habitats (around 15%; *W* = 8100, *P* < 0.001, *n* = 90). In alpine meadows, the community average height was 30 cm, whereas in alpine sandy habitats, it was 15 cm (*W* = 8100, *P* < 0.001, *n* = 90).

### Do pollinator attributes vary across the habitats?

**Figure 3.**
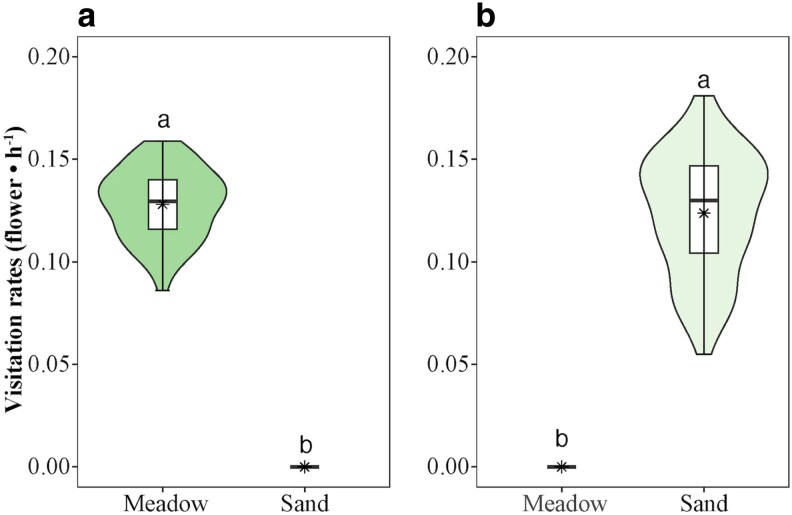
The violin plots show visitation rates of bumble bees (a) and mason bees (b) of *Astragalus purpurinus* between alpine meadows and alpine sandy habitats. Violin plots show the density (width), interquartile range (hinges), and 1.5 times the interquartile range (adjacent lines). The line within the box represents the median of the responses, and the black star within the box represents the mean of the responses. Diﬀerent letters above the boxes denote signiﬁcant diﬀerences among treatments (*P* < 0.05).

The richness and the visitation rates of pollinators varied among the habitats (Fig. 3; see Supplementary Figure S2). *Bombus* was the principal pollinator with high visitation rates in alpine meadows (*W* = 8100, *P* < 0.001, *n* = 90), including *Bombus friseanus*, *B. supremus*, and *B. filchnerae*. In alpine sandy habitats, mason bees served as the principal pollinator with relatively low visitation rates (*W* = 0, *P* < 0.001, *n* = 90).

### Do seed traits vary across the habitats?

**Figure 4.**
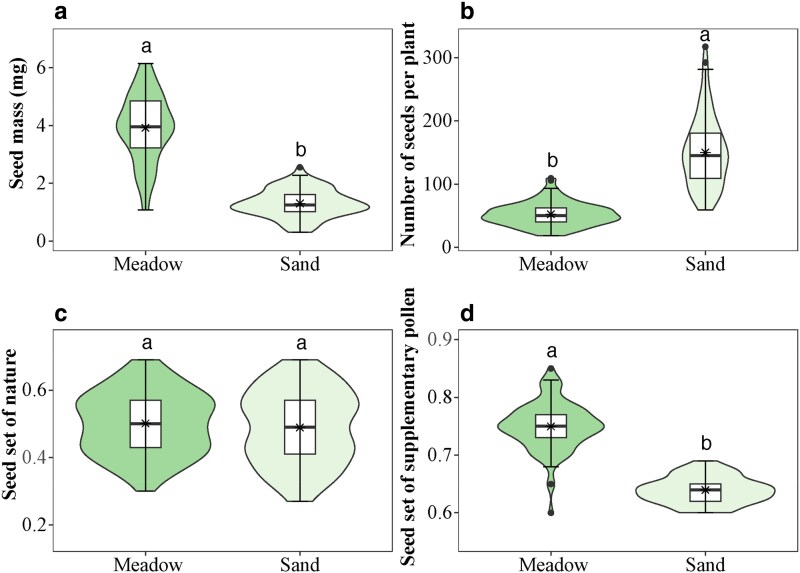
The violin plots show seed mass (a), number of seeds per plant (b), seed set in nature (c), and seed set by supplementary pollen (d) of *Astragalus purpurinus* between alpine meadows and alpine sandy habitats. Violin plots show the density (width), interquartile range (hinges), and 1.5 times the interquartile range (adjacent lines). The line within the box represents the median of the responses, and the black star within the box represents the mean of the responses. Diﬀerent letters above the boxes denote signiﬁcant diﬀerences among treatments (*P* < 0.05).

Small-scale habitat heterogeneity significantly influenced reproductive traits, including seed mass, the number of seeds per plant, and reproductive allocation (Fig. 3; see Supplementary Figure S3). Plants in alpine meadows exhibited larger seeds (*W* = 7882, *P* < 0.001, *n* = 90) and an increased seed set (*W* = 7951, *P* < 0.001, *n* = 90) following hand-pollination compared to plants in alpine sandy habitats. Plants in alpine meadows produced more seeds (*W* = 160, *P* < 0.001, *n* = 90) and had a greater reproductive allocation (*W* = 8100, *P* < 0.001, *n* = 90) compared to those in sandy habitats (see Supplementary Figure S3). However, seed set in nature did not differ significantly between habitats (*W* = 4283, *P* = 0.505, *n* = 90; Fig. 4).

### Do the trait relationships vary among the habitats?

**Figure 5.**
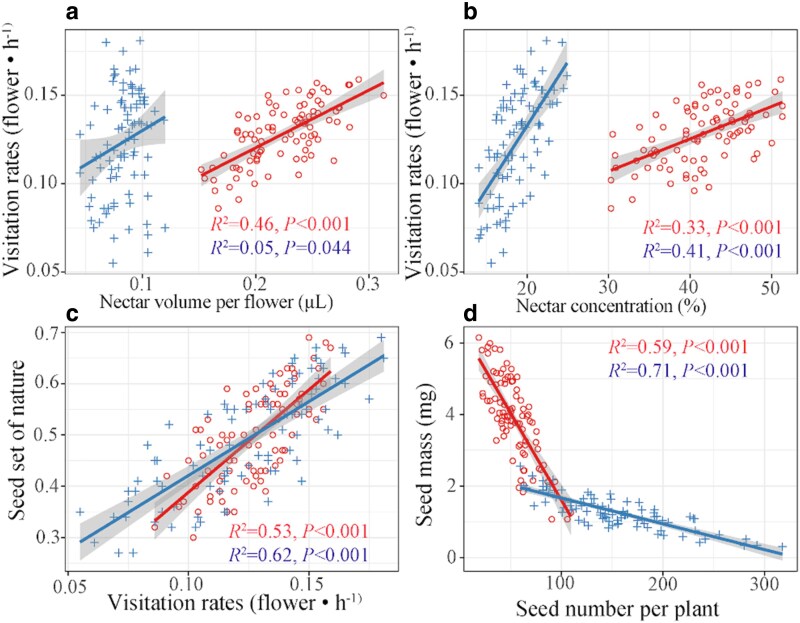
The correlations between visitation rates and nectar volume per flower (a), visitation rates and nectar concentration (b), seed set and visitation rates (c), and seed mass and seed number per plant (d) in alpine meadows and alpine sandy land. The red circles, lines, and words refer to the trait relationships in the alpine meadows, and the blue pluses, lines, and words refer to the trait relationships in the sand habitats.

**Figure 6.**
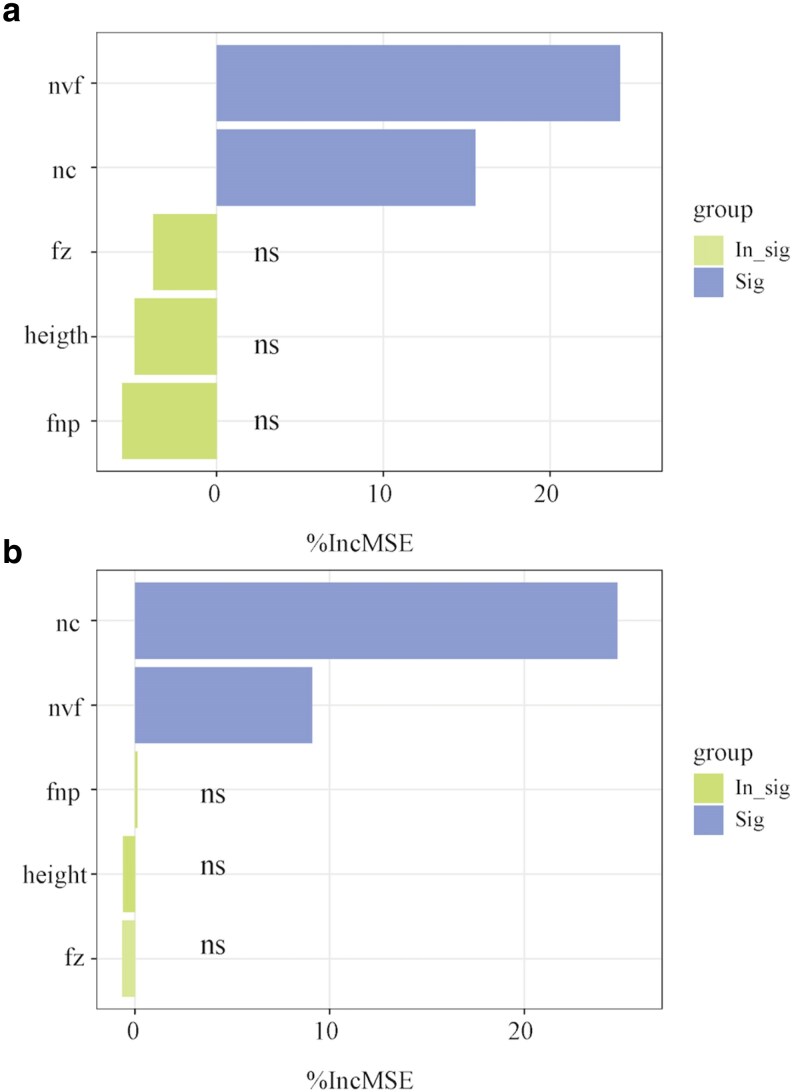
Random forest analysis identified vegetative and flower traits that dominate changes in visitation rates of bumble bees (a) and mason bees (b). * *P* < 0.05, ** *P* < 0.01. ‘nvf’ refers to nectar volume per flower, ‘nc’ refers to nectar concentration, ‘fz’ refers to flower size, ‘height’ refers to plant height, and ‘fnp’ refers to flower number per plant. %IncMSE indicates the increase of the Mean Squared Error when a given variable is randomly permuted. The larger the value of IncMSE, the greater the influence of the characteristic variable on the target. Blue bars indicate statistical significance, and green bars indicate statistical insignificance.

The correlations between the attributes of flora and pollinators varied across habitats (see Supplementary Figure S4). In alpine meadows, we observed a substantial correlation between nectar volume per flower and nectar concentration. However, this relationship was absent in alpine sandy habitats. In both habitats, strong positive correlations were found between pollinator visitation rates and nectar volume per flower. A negative association was observed between seed number and seed mass. Furthermore, a positive relationship between seed set and pollinator visitation rates was found in both alpine meadows and sandy habitats (Fig. 5). The random forest model further indicated that nectar volume per flower and nectar concentration significantly influenced visitation rates of bees in both alpine meadow and sandy habitats (Fig. 6).

Soil moisture, soil temperature, and soil total nitrogen significantly influenced nectar volume per flower (Table 1). Nectar concentration was considerably influenced by soil moisture, soil temperature, soil total phosphorus, and soil total carbon (Table 1). Soil moisture markedly affected flower size and the number of flowers per plant (Table 1). Soil total phosphorus significantly impacted plant height (Table 2).

**Table 1.** Effects of soil properties on flower traits of *Astragalus purpurinus* were analyzed using generalized linear mixed models.

Predictors	Nectar volume per flower			Nectar concentration			Flower size			Number of flowers per plant		
	Estimates	CI	*P*	Estimates	CI	*P*	Estimates	CI	*P*	Estimates	CI	*P*
(Intercept)	−1.98***	−2.04 to −1.92	<0.001	3.33***	3.31 to 3.36	<0.001	0.63***	0.61 to 0.66	<0.001	5.18***	5.12 to 5.25	<0.001
Soil moisture	0.12*	0.01 to 0.22	0.029	0.09*	0.01 to 0.17	0.021	1.12**	1.05 to 1.20	0.001	−0.27*	−0.53 to −0.02	0.036
Soil temperature	−0.12*	−0.24 to −0.01	0.038	−0.12**	−0.20 to −0.04	0.003	0.98	0.91 to 1.04	0.454	−0.09	−0.25 to 0.06	0.247
Total soil nitrogen	0.14*	0.01 to 0.27	0.030	0.10	−0.01 to 0.20	0.065	1.08	0.99 to 1.19	0.081	−0.29	−0.69 to 0.11	0.149
Total soil phosphorus	0.16	−0.00 to 0.32	0.053	0.18**	0.05 to 0.31	0.006	0.97	0.88 to 1.07	0.599	−0.11	−0.38 to 0.16	0.413
Total soil carbon	−0.06	−0.19 to 0.07	0.390	−0.11*	−0.21 to −0.00	0.042	1.04	0.96 to 1.13	0.336	0.04	−0.26 to 0.34	0.796
Random effects												
σ^2^		0.00			20.28			0.01			3722.35	
τ_00_		0.00_plot_			0.00_plot_			0.00_plot_			0.00_plot_	
		0.00_plant_			0.00_plant_			0.00_plant_			0.00_plant_	
ICC		0.61			–			0.05			–	
N		3_plant_			3_plant_			3_plant_			3_plant_	
		10_plot_			10_plot_			10_plot_			10_plot_	
Observations	180			180			180			180		
Marginal *R*^2^/Conditional *R*^2^	0.991/0.996			0.007/NA			0.828/0.837			0.000/NA		

CI, the confidence intervals; σ^2^, variance; τ_00,_ intercept variance; ICC, the intra-class correlation coefficient; N, sample number.

The bold font represents statistical test results that reached a significant level, *P < 0.05 **P < 0.01 ***P  *<* 0.001

**Table 2.** Effects of soil properties on plant height and seed traits of *Astragalus purpurinus* were analyzed using generalized linear mixed models.

Predictors	Plant height			Seed set			Seed mass			Number of seeds per plant		
	Estimates	CI	*P*	Estimates	CI	*P*	Estimates	CI	*P*	Estimates	CI	*P*
(Intercept)	2.39***	2.37 to 2.42	<0.001	−0.71***	−0.75 to −0.66	<0.001	2.25***	2.07 to 2.44	<0.001	4.48***	4.39 to 4.57	<0.001
Soil moisture	0.06	−0.01 to 0.14	0.092	0.23***	0.11 to 0.36	<0.001	0.94	0.75 to 1.19	0.626	−0.07	−0.41 to 0.28	0.707
Soil temperature	−0.08	−0.15 to 0.00	0.055	0.10	−0.02 to 0.22	0.095	0.89	0.71 to 1.11	0.292	−0.06	−0.27 to 0.16	0.611
Total soil nitrogen	0.04	−0.06 to 0.13	0.465	0.00	−0.17 to 0.18	0.990	1.30	0.93 to 1.81	0.127	−0.47	−1.00 to 0.07	0.087
Total soil phosphorus	0.20**	0.08 to 0.32	0.001	−0.25**	−0.43 to −0.06	0.009	1.53*	1.09 to 2.16	0.015	−0.25	−0.61 to 0.11	0.171
Total soil carbon	−0.02	−0.11 to 0.08	0.748	0.13	−0.03 to 0.28	0.105	0.82	0.61–1.10	0.189	0.19	−0.21 to 0.58	0.357
Random effects												
σ^2^		2.66			0.01			0.11			1689.94	
τ_00_		0.00_plot_			0.00_plot_			0.00_plot_			0.00_plot_	
		0.00_plant_			0.00_plant_			0.00_plant_			0.00_plant_	
ICC								0.03				
*N*		3_plant_			3_plant_			3_plant_			3_plant_	
		10_plot_			10_plot_			10_plot_			10_plot_	
Observations	180			180			180			180		
Marginal *R*^2^/Conditional *R*^2^	0.046/NA			0.276/NA			0.730/0.739			0.000/NA		

CI, the confidence intervals; σ^2^, variance; τ_00,_ intercept variance; ICC, the intra-class correlation coefficient (ICC); N, sample number.

The bold font represents statistical test results that reached a significant level. *P < 0.05, **P < 0.01, ***P < 0.001.

### Are differences in pollinator visitation rates due to variation in plant community or nectar concentration?

Plants in alpine meadow habitats exhibited high visitation rates of bumble bees when transplanted in alpine meadow and sandy habitats (see Supplementary Figure S5a, b). Similarly, plants in alpine sandy habitats exhibited a high visitation rate of mason bees when transplanted in both alpine meadow and sandy habitats (see Supplementary Figure S5c, d).

**Figure 7.**
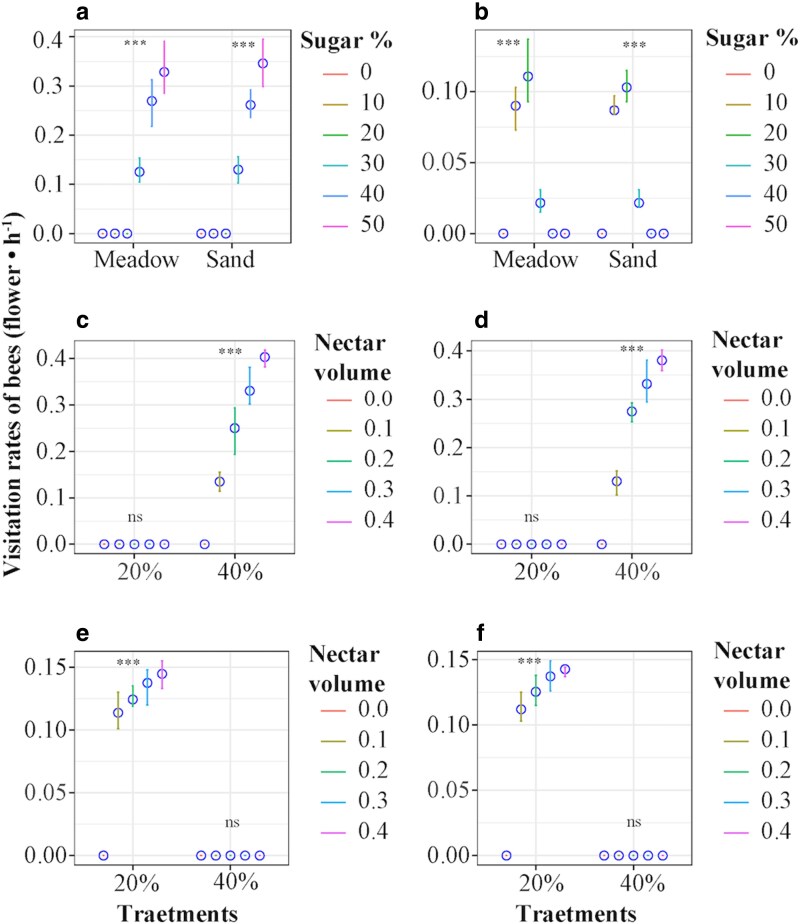
The figures show the visitation rate of bumble bees (a) and mason bees (b) at the nectar concentration (0%–50% sugar) addition treatment, the visitation rate of bumble bees in alpine meadows (c) and in alpine sandy habitats (d), as well as the visitation rate of mason bees in alpine meadows (e) and in alpine sandy habitats (f) at the nectar volume (0–0.4 µl) addition treatment. The blue circle represents the mean of the responses. The line represents the standard error. The star denotes signiﬁcant diﬀerences among treatments (*P* < 0.05).

The nectar addition experiment revealed that bumble bees chose flowers with high nectar concentrations (40%–50% sugar; Fig. 7a) but mason bees favored flowers with low nectar concentrations (10%–20% sugar; Fig. 7b). The visitation rates of both bees exhibited a positive correlation with nectar volume per flower (Fig. 7c–f).

## Discussion

Our results demonstrated that meadow populations and sandy populations of *A. purpurinus* have different vegetative and floral traits, pollinator attributes, and reproductive strategies. In the meadow populations, elevated individual height and nectar incentives had increased bumble bee visitation, correlating with the production of fewer and larger seeds. However, sandy populations had reduced individual height and nectar incentives, which enticed mason bees and was associated with the production of a greater number of small seeds. These two distinct mechanisms enable *A. purpurinus* to adapt to differences in their respective habitats.

Small-scale habitat heterogeneity modified various plant traits, including plant biomass and plant height, flower morphology and display, floral rewards, and flowering phenology (Aizen and Vazquez 2006, Hass *et al*. 2018, Battle *et al*. 2021, Berthon *et al*. 2021, Bueno and Iglesias 2021). In this study, although the sandy habitats were situated <10 m from the meadows, soil moisture and nutrient levels differed significantly. Alpine meadow soil had higher nutrients and moisture than alpine sandy soil (Gao *et al*. 2016, Wang *et al*. 2023). Plants in alpine meadows exhibited higher nectar rewards and increased individual height (Fig. 2; see Dai *et al*. 2022). In contrast, plants in alpine sandy habitats exhibited reduced nectar production, lower nectar concentrations (Fig. 2), and decreased individual height. It is important to note that nectar production often incurs significant nutritional and carbohydrate costs (Heinrich and Raven 1972, Pyke 1991, Mu *et al*. 2015). Plants with nectar-deficient flowers may thus gain a competitive advantage by reallocating resources to enhance other vegetative or floral traits. Previous studies report a strong inverse relationship between nectar production and both the number of flowers per plant and flower size (Pleasants and Chaplin 1983, Mu *et al*. 2014). Our analysis confirmed this trade-off, revealing that diminished nectar production and concentrations were associated with increased flower and inflorescence number. Alpine meadow populations produced flowers with abundant, high-quality nectar but fewer flowers and inflorescences. In contrast, alpine sandy populations increased flower and inflorescence number while reducing both nectar amount and quality. This suggests that small-scale habitat variability drives floral trait trade-offs.

The diversity of habitat-mediated traits enables plants to adapt to varying habitats. In alpine meadows, populations producing abundant nectar attract pollinators, promoting pollination and increasing seed set (Dai *et al*. 2022, Wang *et al*. 2024). *Astragalus purpurinus—*a rare species with <5% relative abundance in the meadows*—*faced competition for pollinators from co-flowering species like *Saussurea nigrescens* and *S. stella* (Mu *et al*. 2014, Su *et al*. 2022). Bumble bees prefer high-nectar rewards due to their larger body and high metabolic demands (Biernaskie and Gegear 2007, Frasnelli *et al*. 2021). Alpine meadow population allocated resources to increased height (intensifying light competition), and the resulting carbon surplus enabled significant nectar rewards (Heinrich and Raven 1972, Pyke 1991, Mu *et al.* 2015), attracting bumble bees and improving pollination and seed set. In nutrient- and moisture-limited alpine sandy habitats, plants expend energy to withstand arid conditions while allocating relatively scarce resources to reproduction. Consequently, they produce less-concentrated nectar in smaller volumes (Waser and Price 2016, Mueller *et al*. 2020), attracting mason bees—which prefer low-sugar flowers (Ahrenfeldt *et al*. 2019)—ensuring adequate pollination. Simultaneously, *A. purpurinus* in sandy habitats invests energy in producing more flowers and inflorescences, securing sufficient seed production.

A trade-off between seed number and seed mass is common, which influences plant adaptation to diverse habitats (Harper *et al*. 1970, Greene and Johnson 1993, Guo *et al*. 2000). Our study demonstrated that the quality and quantity of seeds varied among different habitats. The population in alpine meadows produced fewer, larger seeds. Larger seeds provide more energy for germination, enhancing seedling competitiveness and establishment success (Jakobsson and Eriksson 2000). In sandy habitats, however, populations produced a larger number of small seeds, which facilitated long-distance dispersal and colonization. *Astragalus purpurinus* has evolved two distinct reproductive strategies to adapt to small-scale habitat heterogeneity.

The quantity and quality of floral rewards affect the diversity and choice of pollinators (Fowler *et al.* 2016, Su *et al*. 2022). For example, bumble bees prefer flowers with higher nectar concentrations (Roubik *et al*. 1995, Cnaani *et al*. 2006). *A. purpurinus* was pollinated by two distinguishable pollinators: bumble bees in alpine meadows and mason bees in alpine sandy habitats. Pollinators exhibit differentiation across small-scale habitat heterogeneity due to the varying concentrations and quantities of nectar provided by flowers. Our nectar addition experiment further demonstrated that bumble bee visitation frequency significantly increased when nectar concentration exceeded 30% but declined dramatically below 30%. Conversely, mason bee visitation peaked at 10%–20% nectar concentration, aligning with findings that mason bees prefer strawberry flowers with 17%–34% sugar content (Ahrenfeldt *et al*. 2019). Overall, the frequency of bee visits to flowers positively correlated with increased nectar volume. Our findings also showed that bumble bees preferred flowers with a nectar concentration of 40%, while mason bees favored those with 20%. Although we observed that both mason bees and bumble bees carried pollen on their bodies, both bees appear to gather pollen passively. They invest considerable time foraging for nectar.

Community composition also affects pollinator visitation rates (Kelly and Elle 2020, Allen-Perkins *et al*. 2025). Our data demonstrated that populations from alpine meadows had a high visitation rate of bumble bees, irrespective of their location in alpine meadows or sandy habitats. Similarly, the populations from alpine sandy habitats had the greatest frequency of floral visits by mason bees, irrespective of their location in alpine meadows or sandy habitats. The findings suggest that intrinsic floral traits outweigh community context in determining pollinator preferences. Flower scent is a crucial factor in pollinator behavior (Farré-Armengol *et al*. 2016). However, our findings indicated that neither bumble bees nor mason bees exhibited a preference for flowers treated with 0.2 µl purified water, implying that flower scent is not associated with pollinator attributes in this context.

## Conclusion

In summary, our findings indicate that *A. purpurinus* exhibits different vegetative and floral traits, pollinator richness and visitation rates, as well as reproductive strategies within small-scale habitat heterogeneity. These two distinct mechanisms enable plants to adapt to various environments. Our findings provide new insights into how small-scale habitat heterogeneity influences plant reproductive strategies and pollinator attributes.

## Supplementary Material

plaf052_Supplementary_Data

## Data Availability

Raw data and R code are available online at https://zenodo.org/records/17054532.
