# Supplementary material for: Plant reproductive strategies and pollinator attributes differ in small-scale habitat heterogeneity
Source: AoB Plants. 2025 Sep 17;17(5):plaf052. doi: 10.1093/aobpla/plaf052 (PMC12480739; doi:10.1093/aobpla/plaf052)

Running head: habitat impacts plant-pollinator interactions

Plant reproductive strategies and pollinator attributes differ in small-scale habitat heterogeneity

Dongzhou Deng[1], Juanli Chen[2], Li He[1], Dawei Li[2], Dechao Chen[1], Wuxian Yan[1], Junpeng Mu[2*]

[1] *Ecological Restoration and Conservation on Forest and Wetland Key Laboratory of Sichuan Province, Sichuan Academy of Forestry Sciences, Chengdu 610081, China*

[2] *Ecological Security and Protection Key Laboratory of Sichuan Province, Mianyang Normal University, Mianyang 621000, China*

*Correspondence author's e-mail address: gbmujp@163.com

Figure S1. The violin plots show soil properties between alpine meadows and alpine sandy habitats. Soil daily average temperature (a), soil moisture (b), soil total nitrogen (c), soil total phosphorus (d), and soil carbon (e). Violin plots show the density (width), interquartile range (hinges), and 1.5 times the interquartile range (adjacent lines). The line within each box represents the median of the responses, and the black star within each box represents the mean of the responses. Different letters above the boxes denote significant differences among treatments ($p < 0.05$).

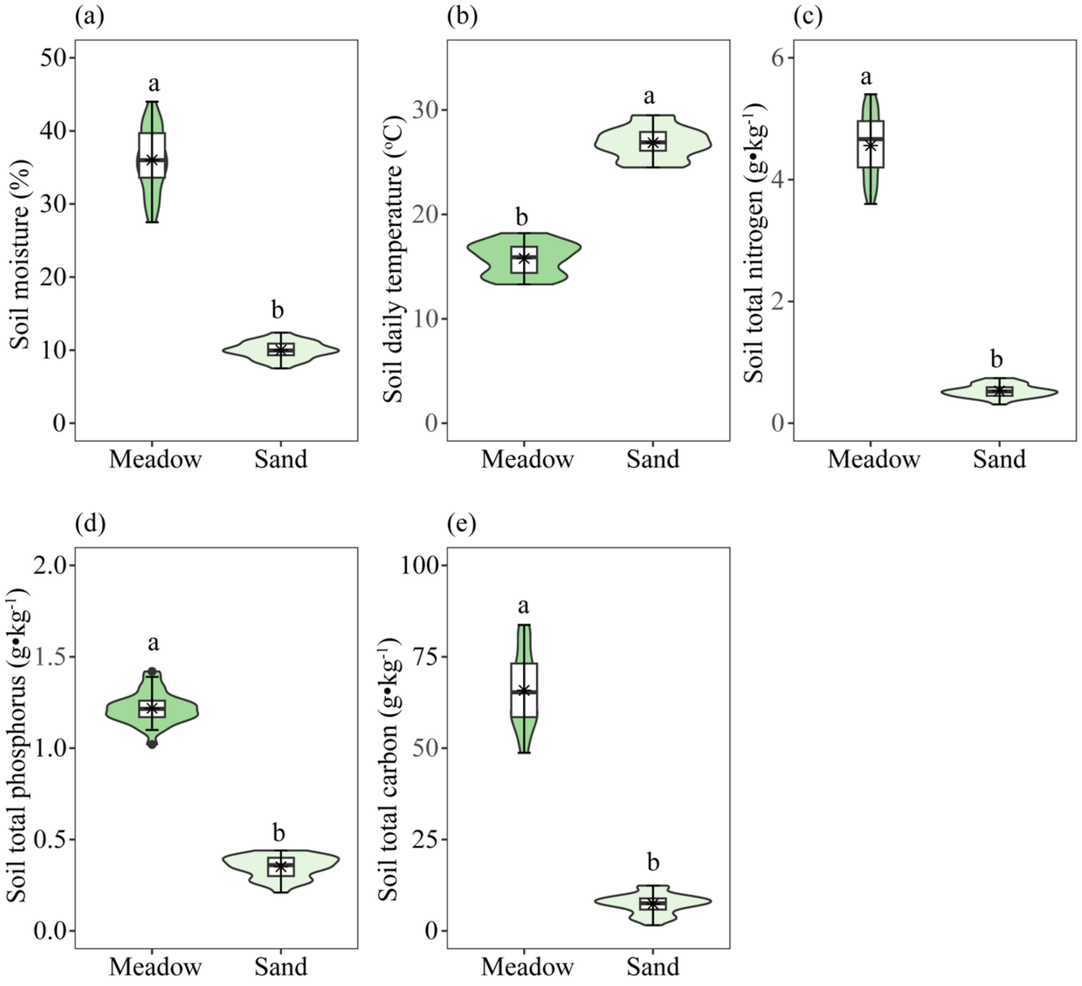

Figure S2. PCoA (principal coordinates analysis) analysis of variation based on the plant traits and pollinator visitation rate of alpine meadows and alpine sandy land. The two orthogonal axes explain 89.41% and 7.85% of the variance, respectively.

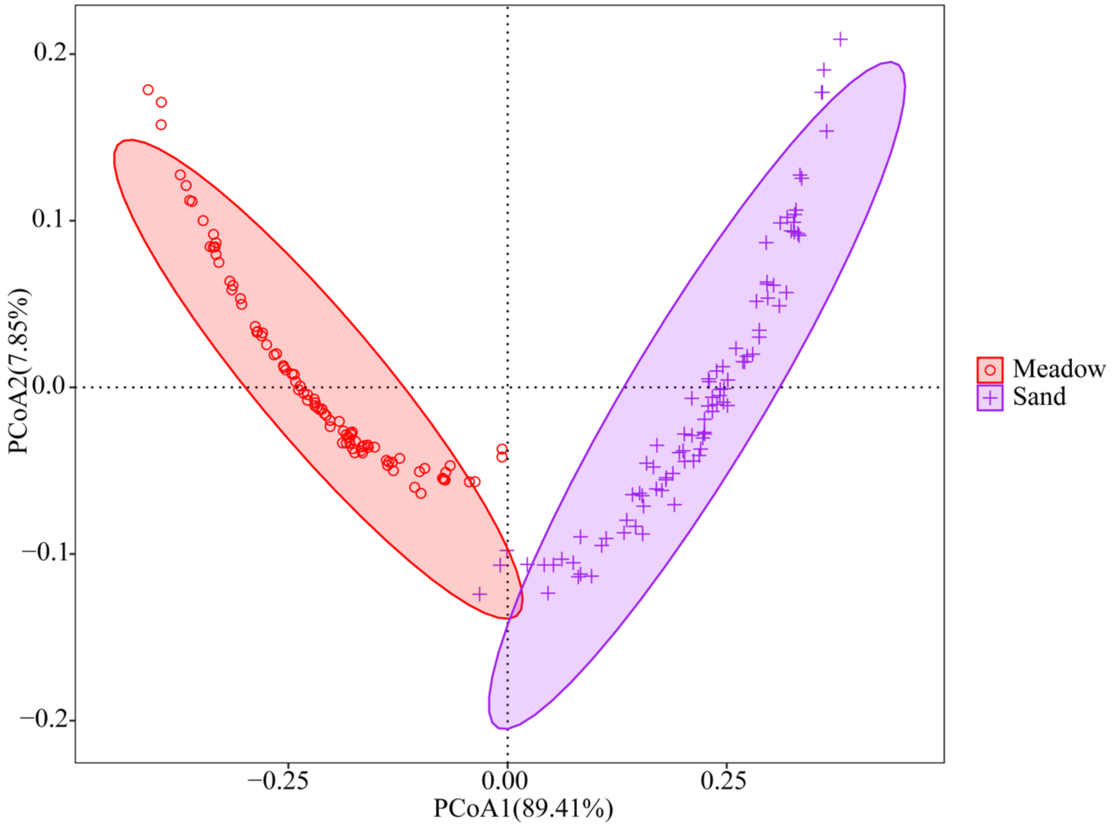

Figure S3. The violin plots show reproduction allocation between alpine meadows and alpine sandy habitats. Violin plots show the density (width), interquartile range (hinges), and 1.5 times the interquartile range (adjacent lines). The line within each box represents the median of the responses, and the black star within each box represents the mean of the responses. Different letters above the boxes denote significant differences among treatments ($p < 0.05$).

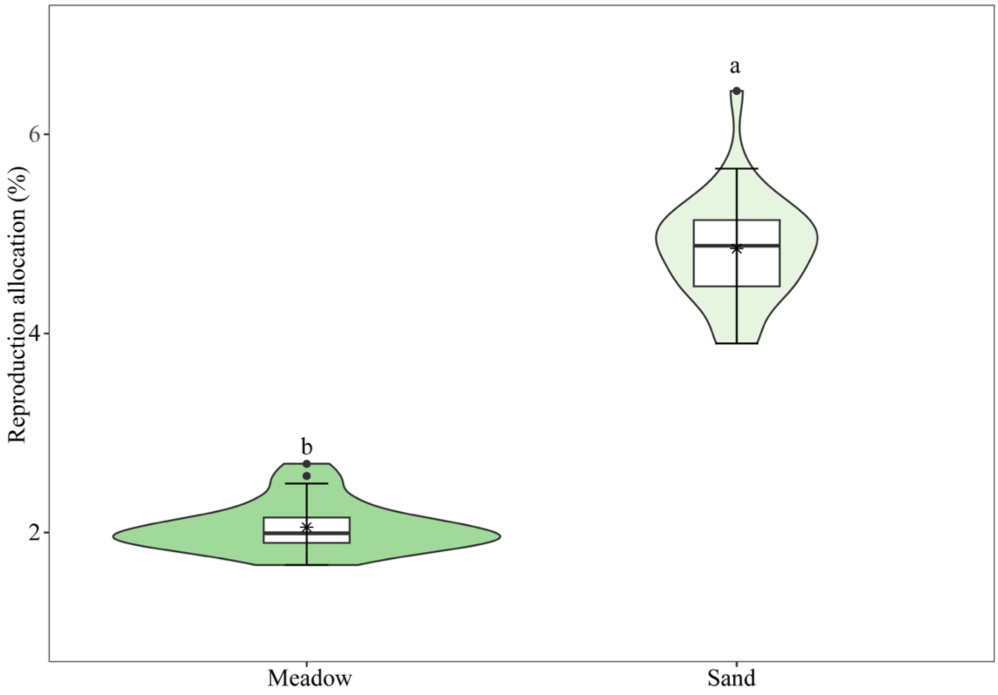

Figure S4. Pearson correlations among the plants, pollinator visitation rates on the meadows (a), and sandy habitats (b). $^{*}p < 0.05$, $^{**}p < 0.01$, $^{***}p < 0.001$. The red line represents a positive relationship, and the green line represents a negative relationship. The term "vr" refers to the visitation rates of pollinators, and "snp" refers to the seed number per plant. The terms "nvf" refer to the nectar volume per flower, "nc" refers to the nectar concentration, "fz" refers to the flower size, "fninf" refers to the number of flowers per inflorescence, "infn" refers to the number of inflorescences per plant, "fnp" refers to the number of flowers per plant, "ssrn" refers to the seed set in nature, "nvf" refers to the seed set with supplementary pollen, and "sm" refers to the seed mass.

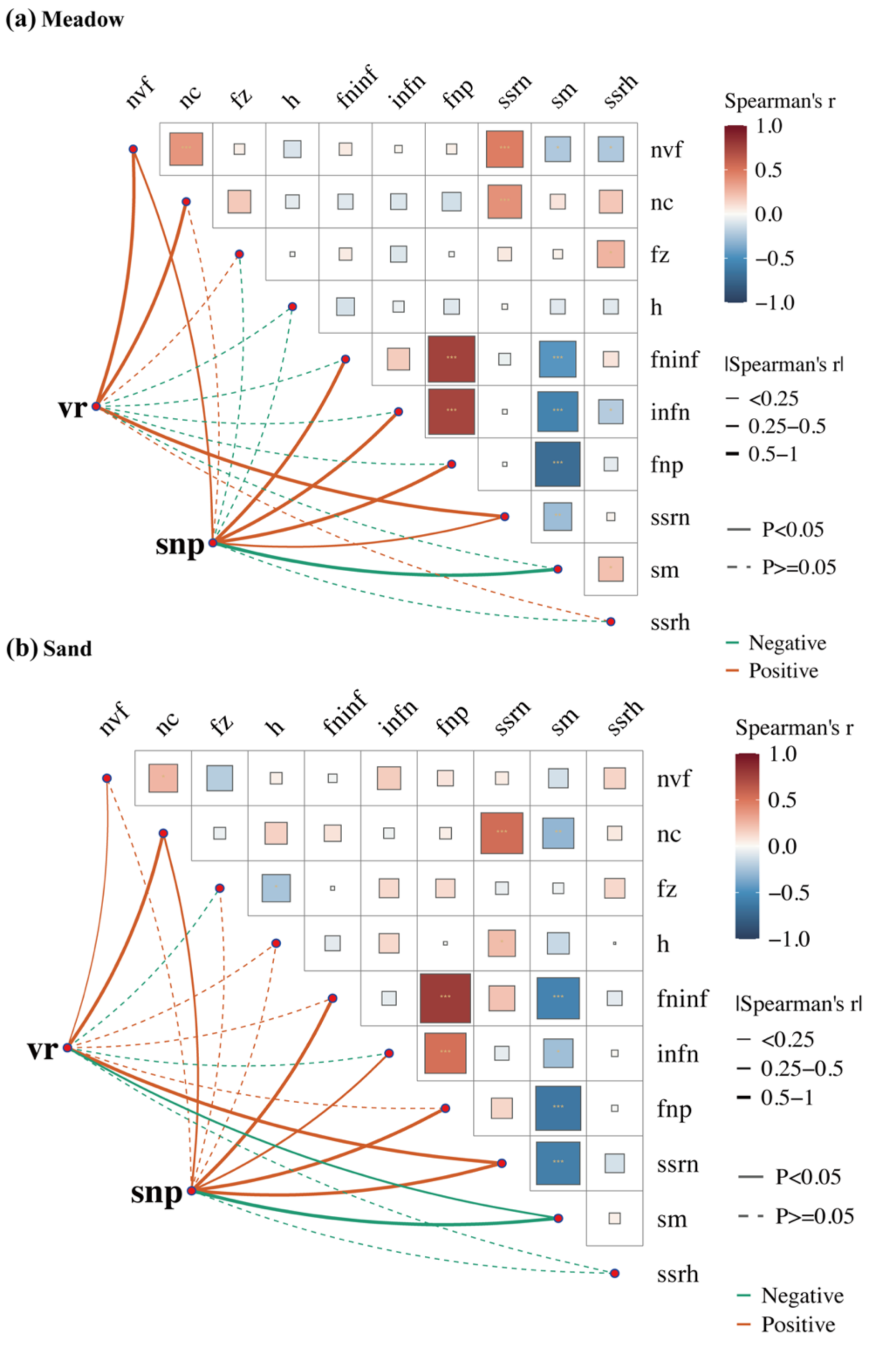

Figure S5. The violin plots display visitation rates of bumble bees to plants from alpine meadow and alpine sandy habitats transplanted into the meadow habitats (a) and sandy habitats (b), as well as visitation rates of mason bees to plants from alpine meadow and alpine sandy habitats that were transplanted into the meadow habitats (c) and sandy habitats (d). Violin plots illustrate the data density (width), interquartile range (hinges), and 1.5 times the interquartile range (adjacent lines). The line within each box represents the median of the responses. Different letters above the boxes indicate significant differences among treatments ($p < 0.05$). P_{meadow} refers to the plants from the meadow habitats, and P_{sandy} refers to the plants from the sandy habitats.

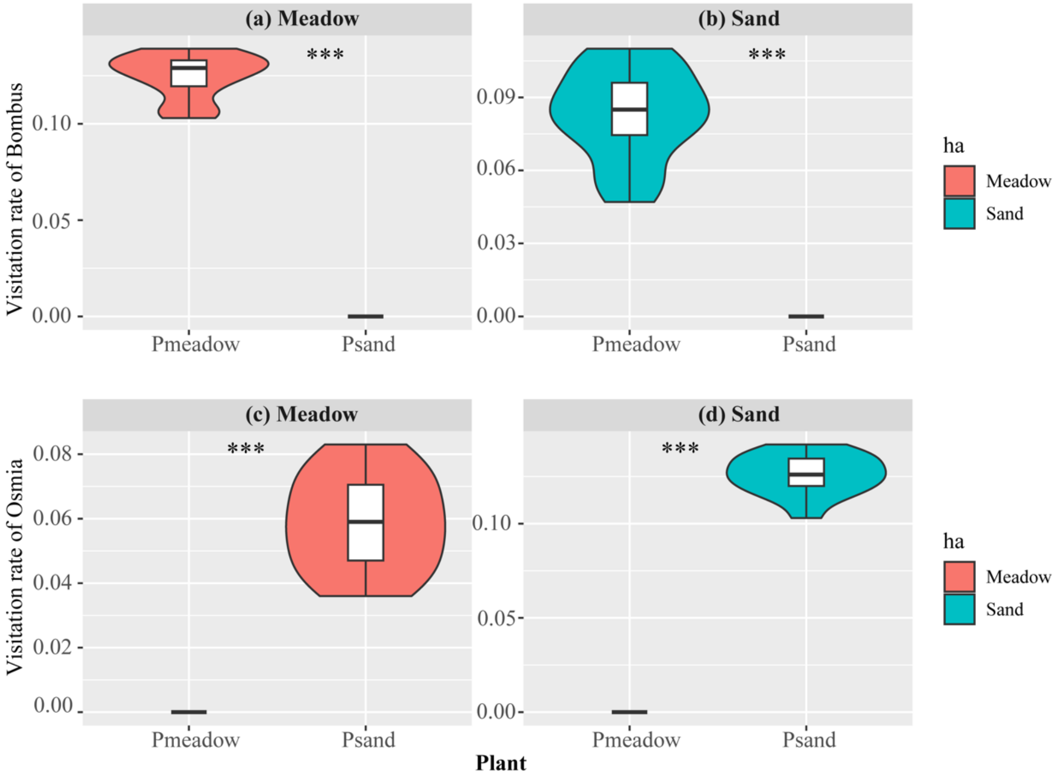

Supplement: plaf052_Supplementary_Data [file plaf052_supplementary_data.zip › Supplementary information.pdf]
